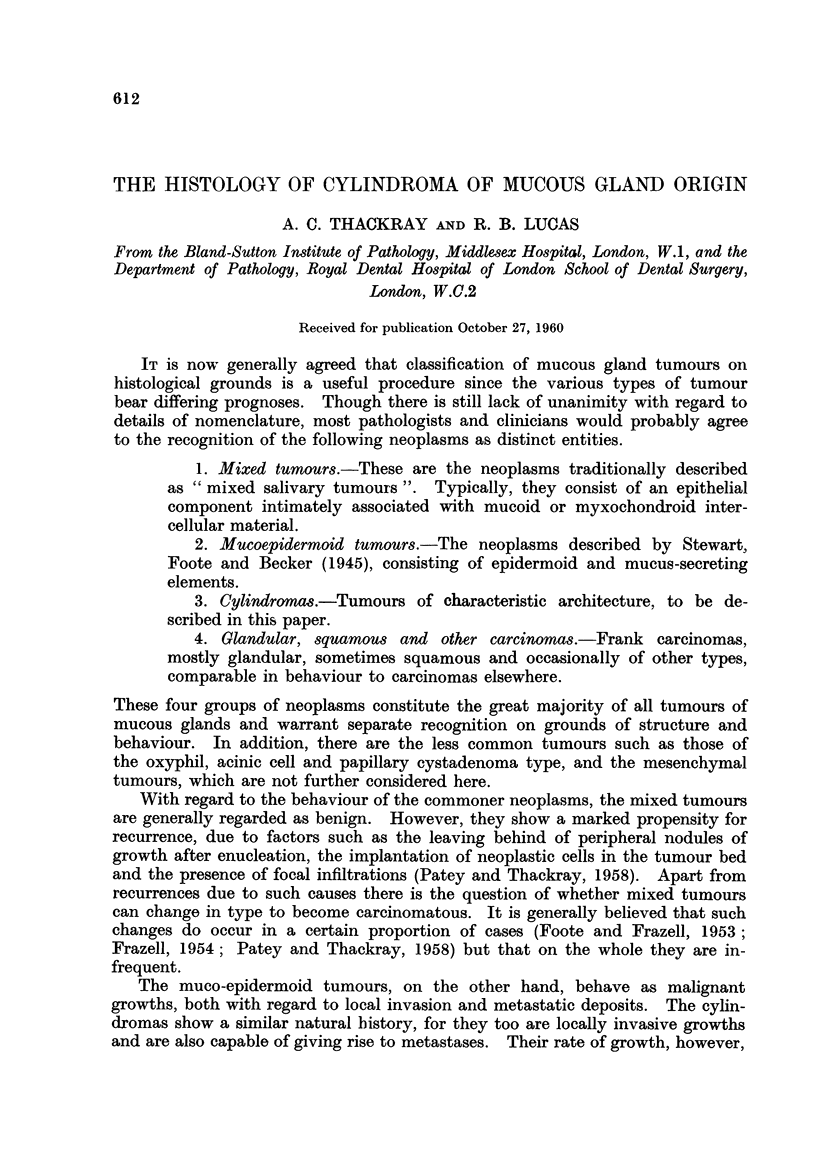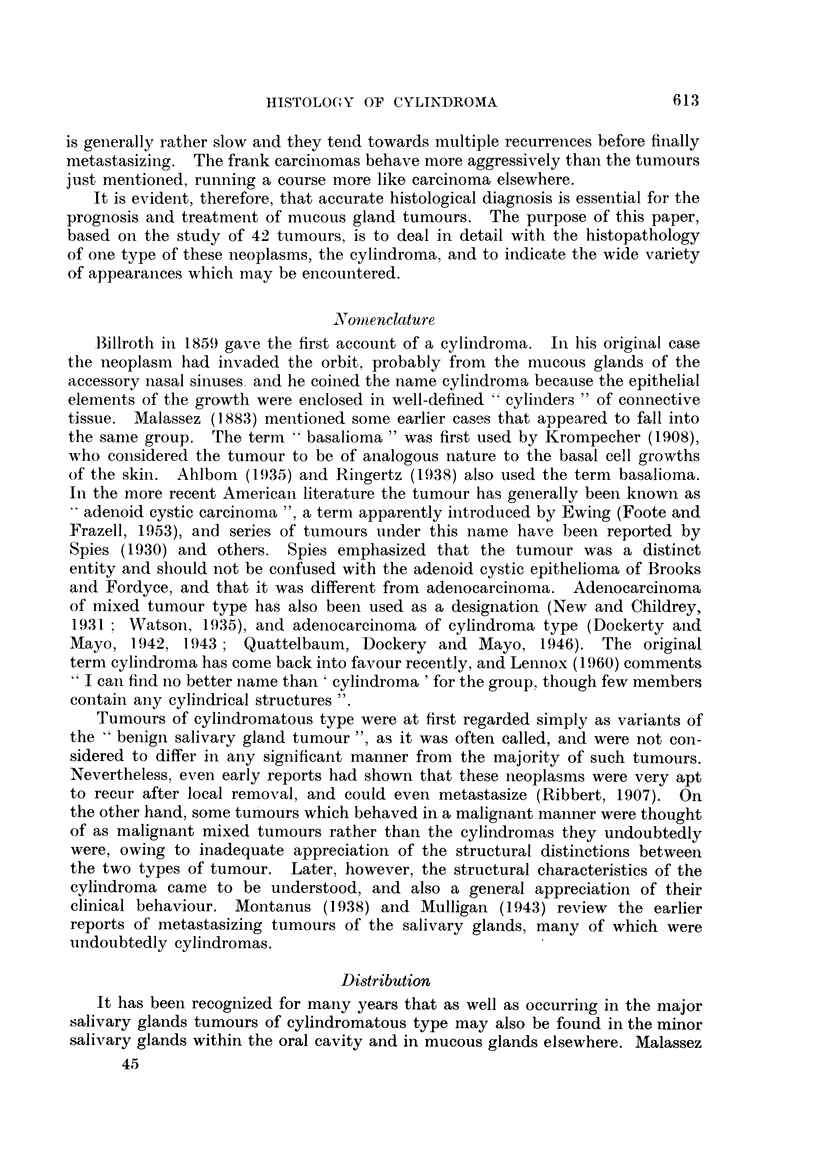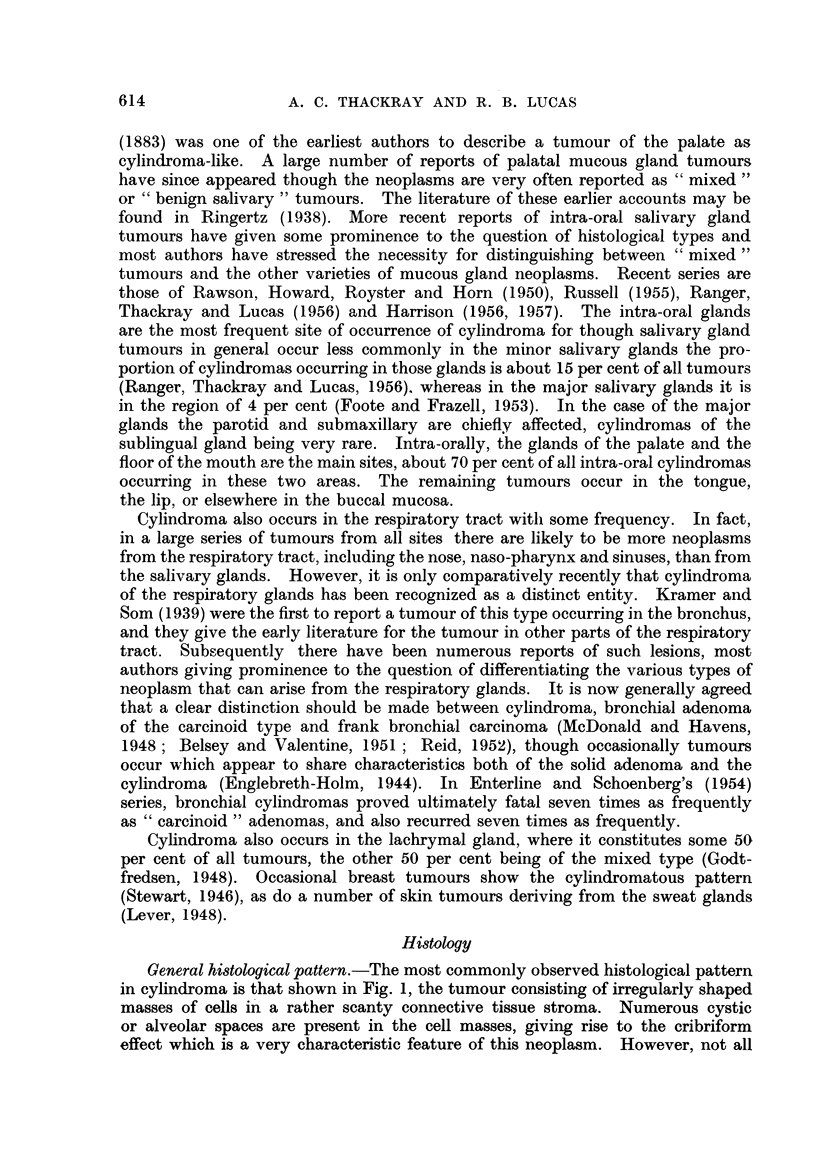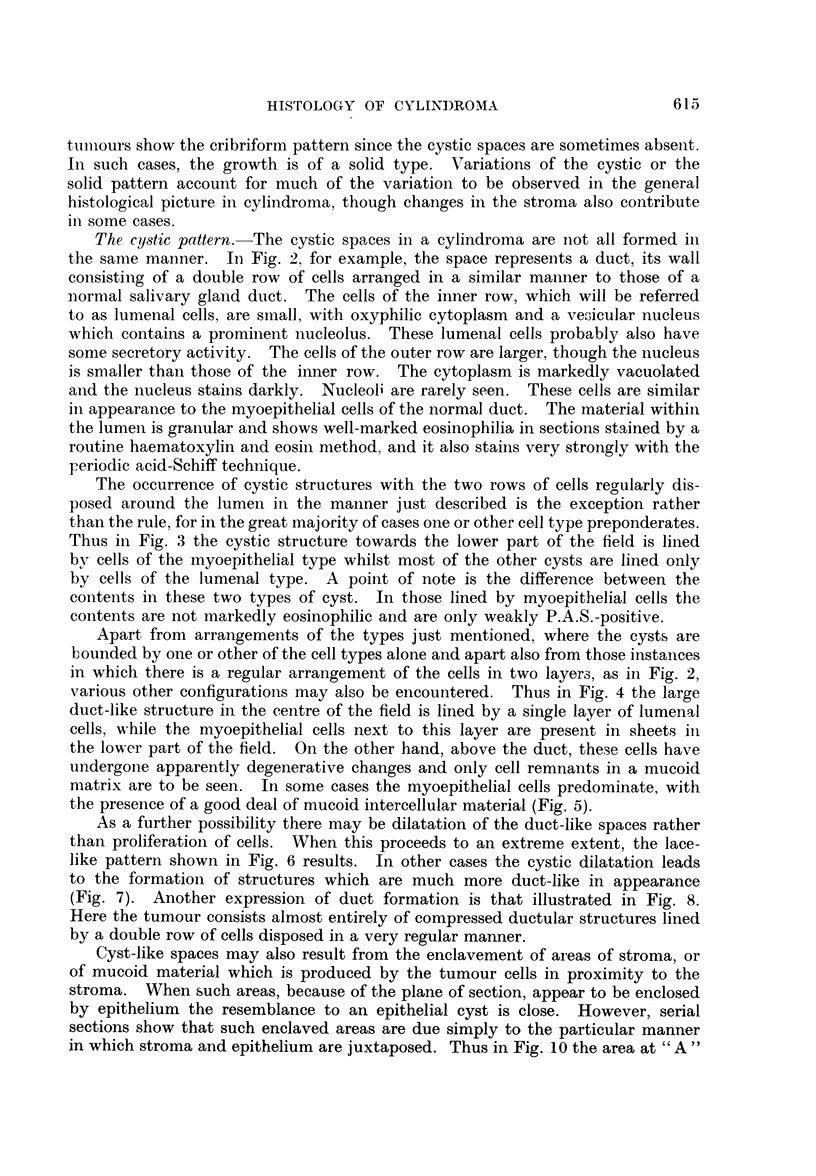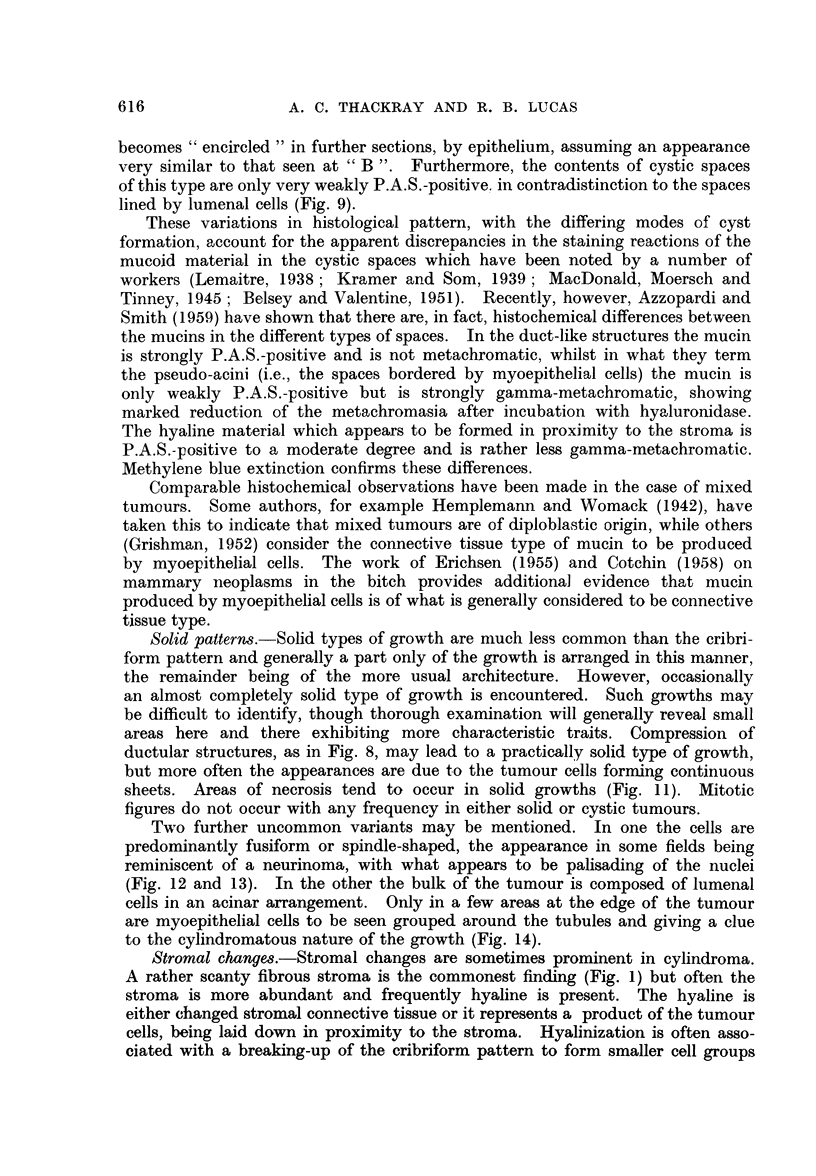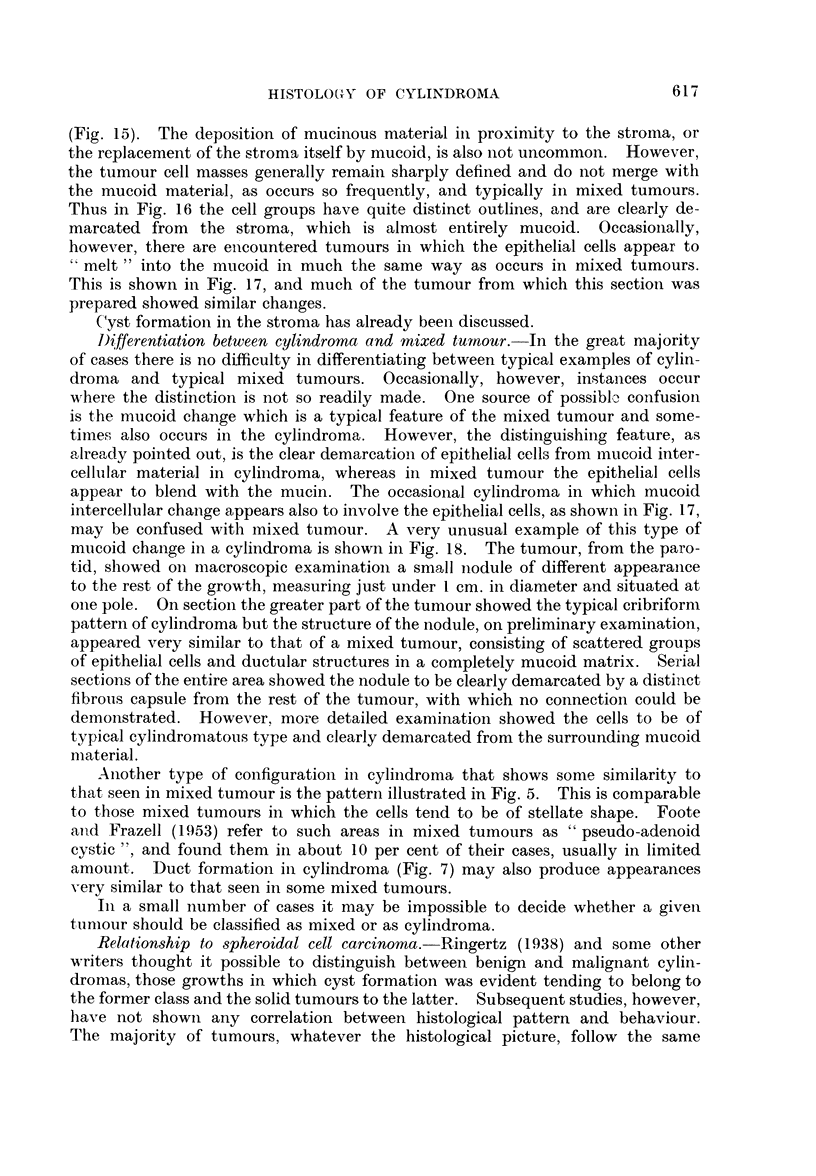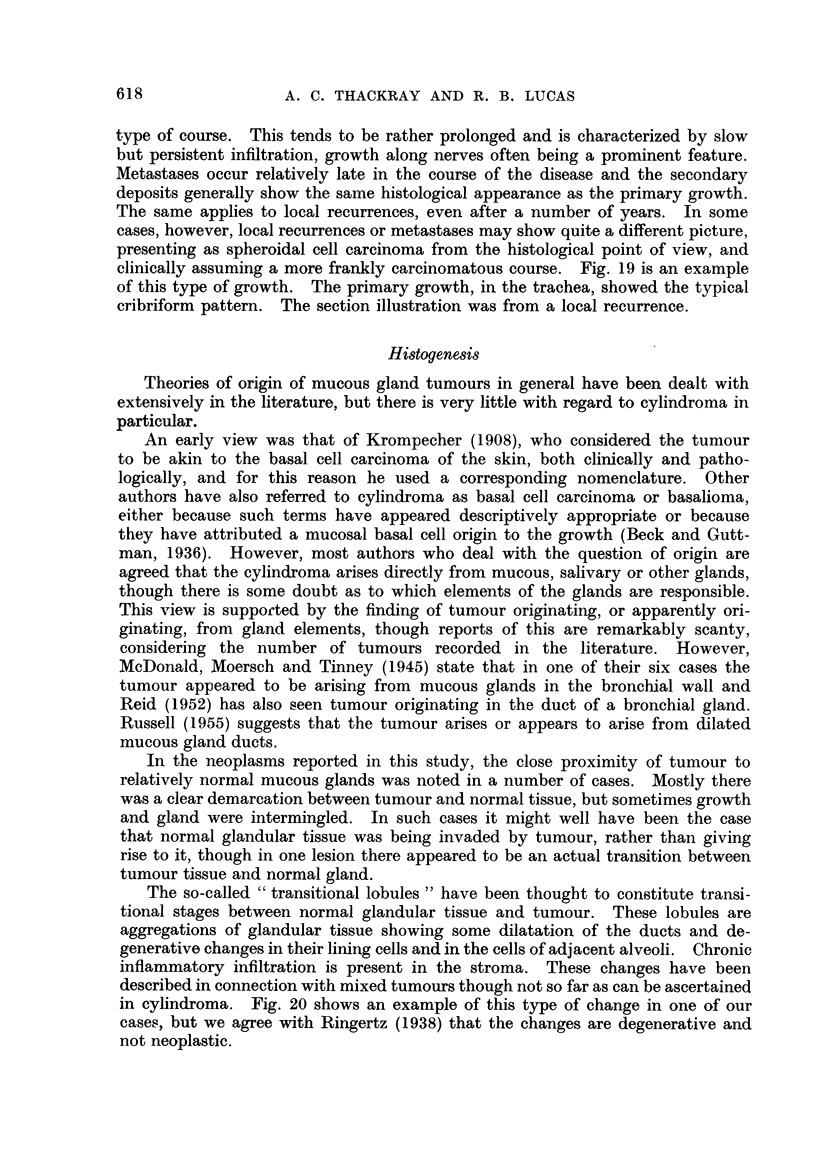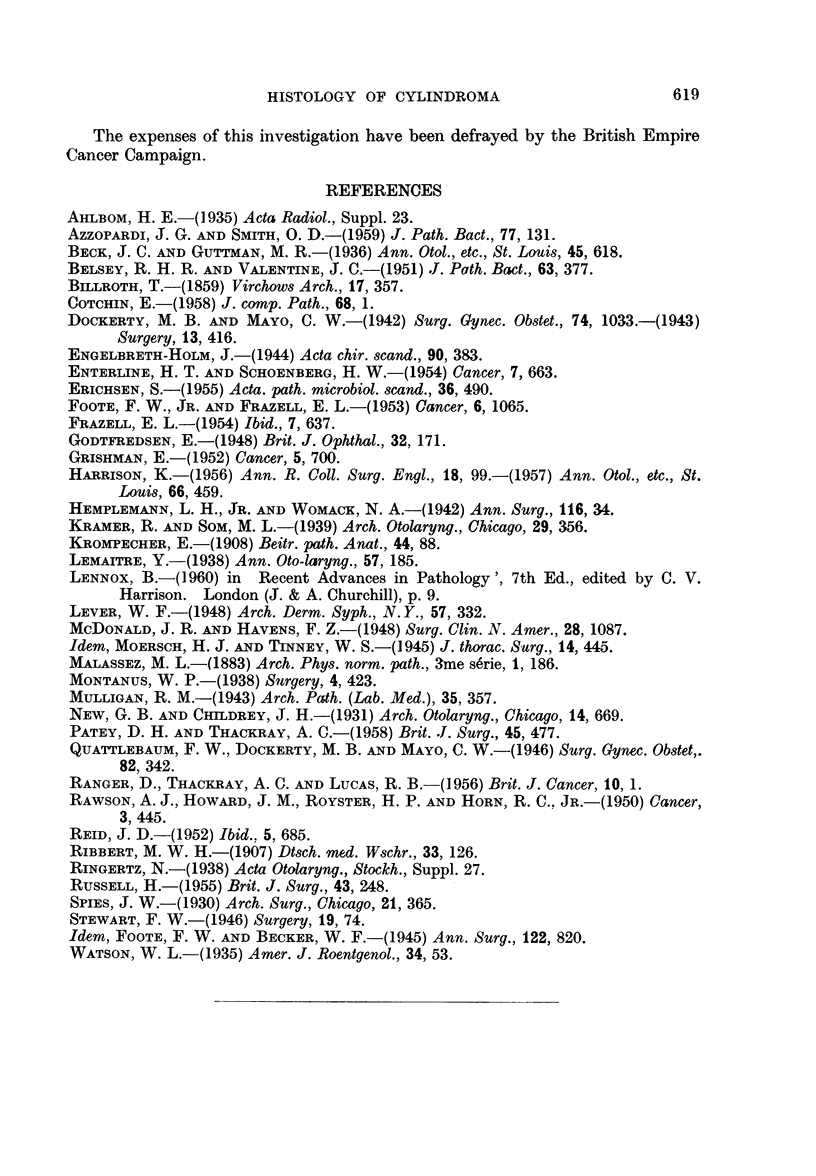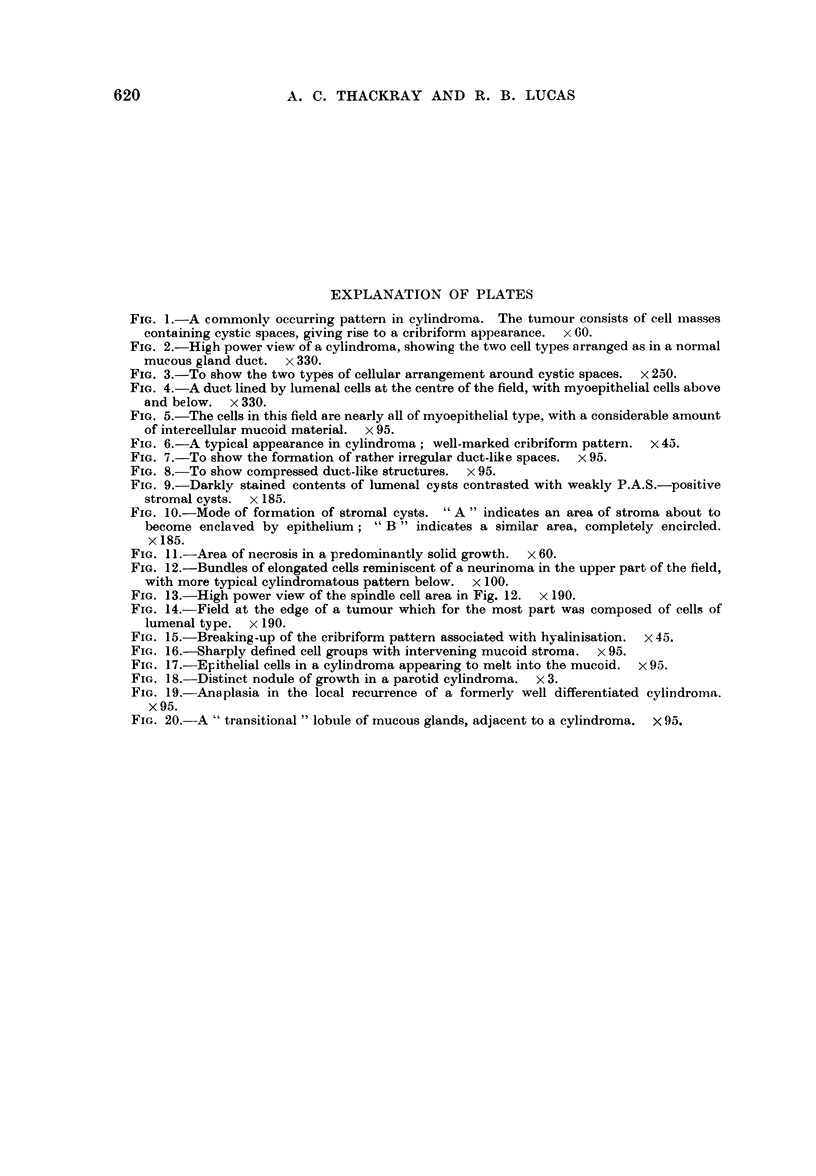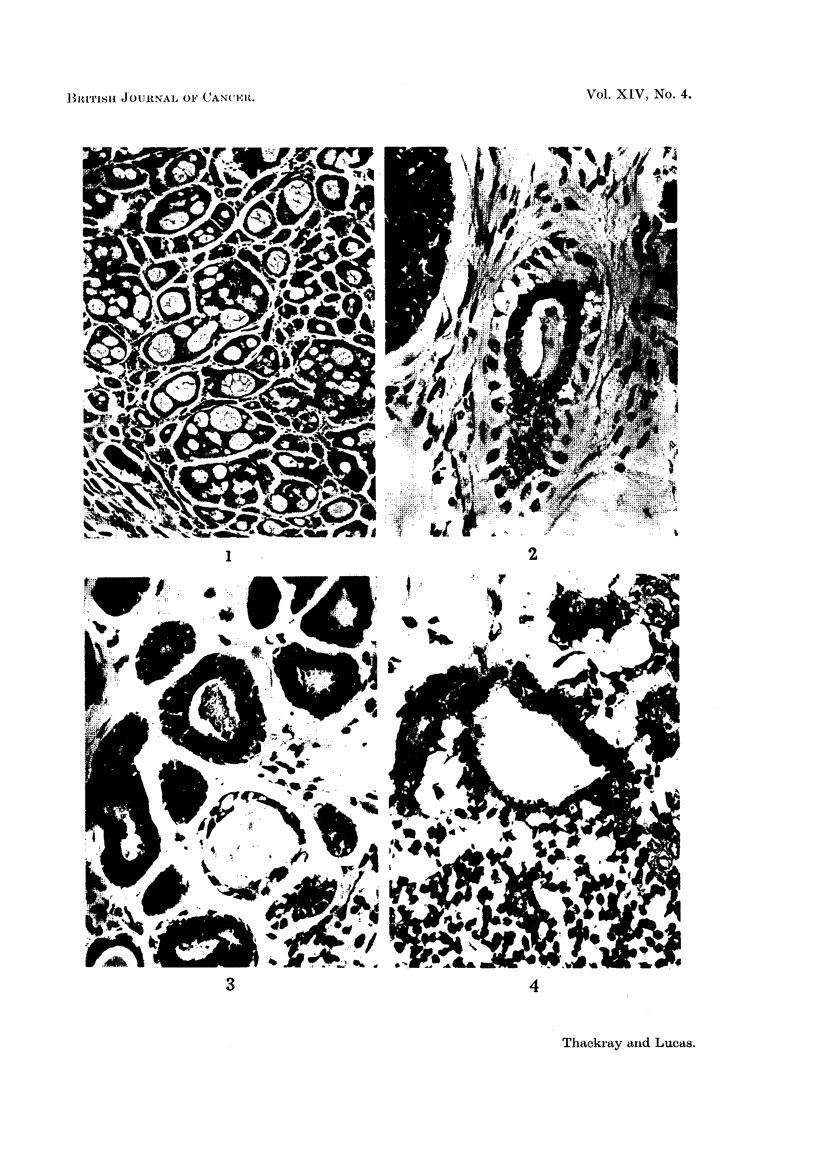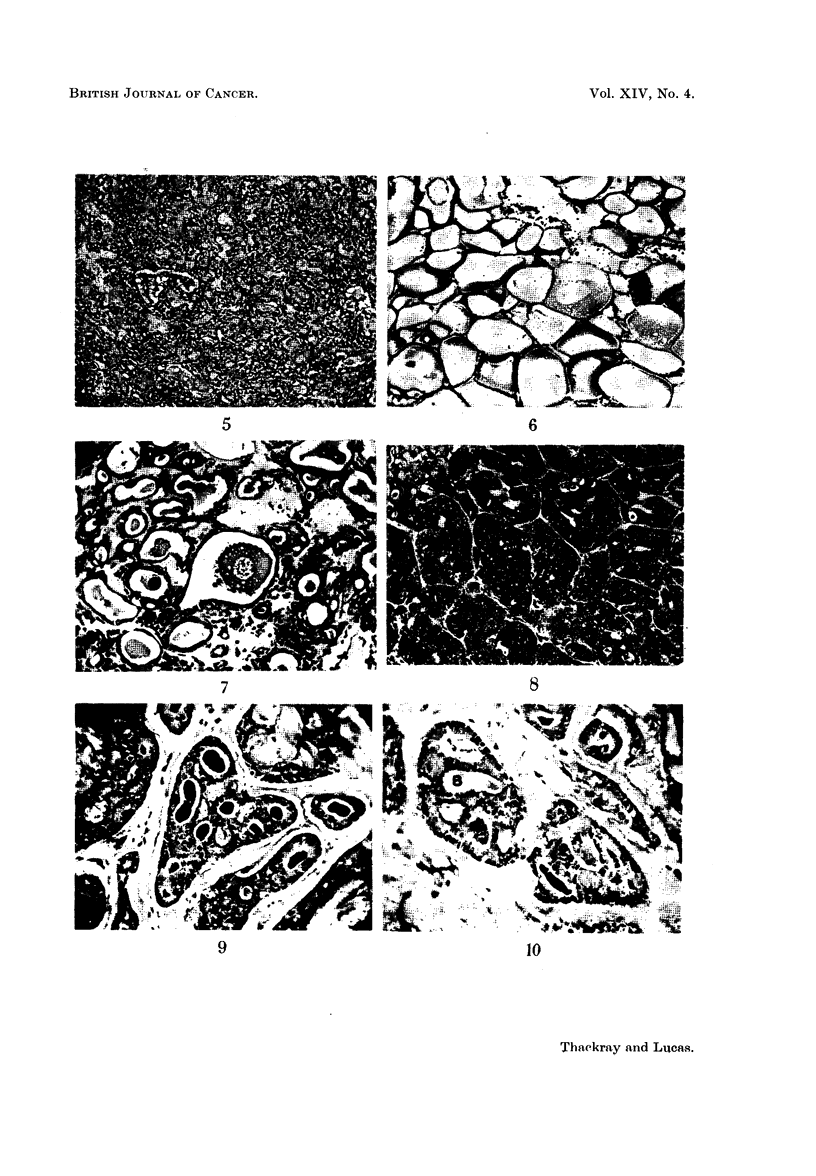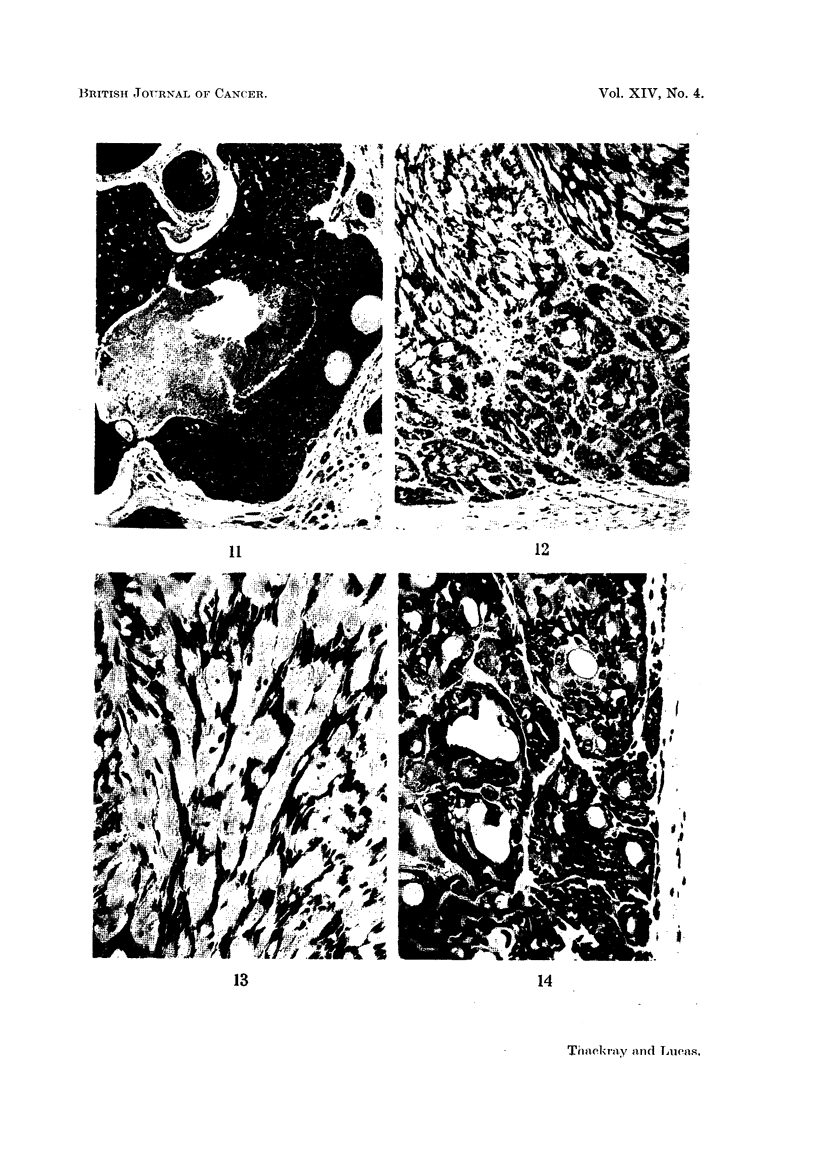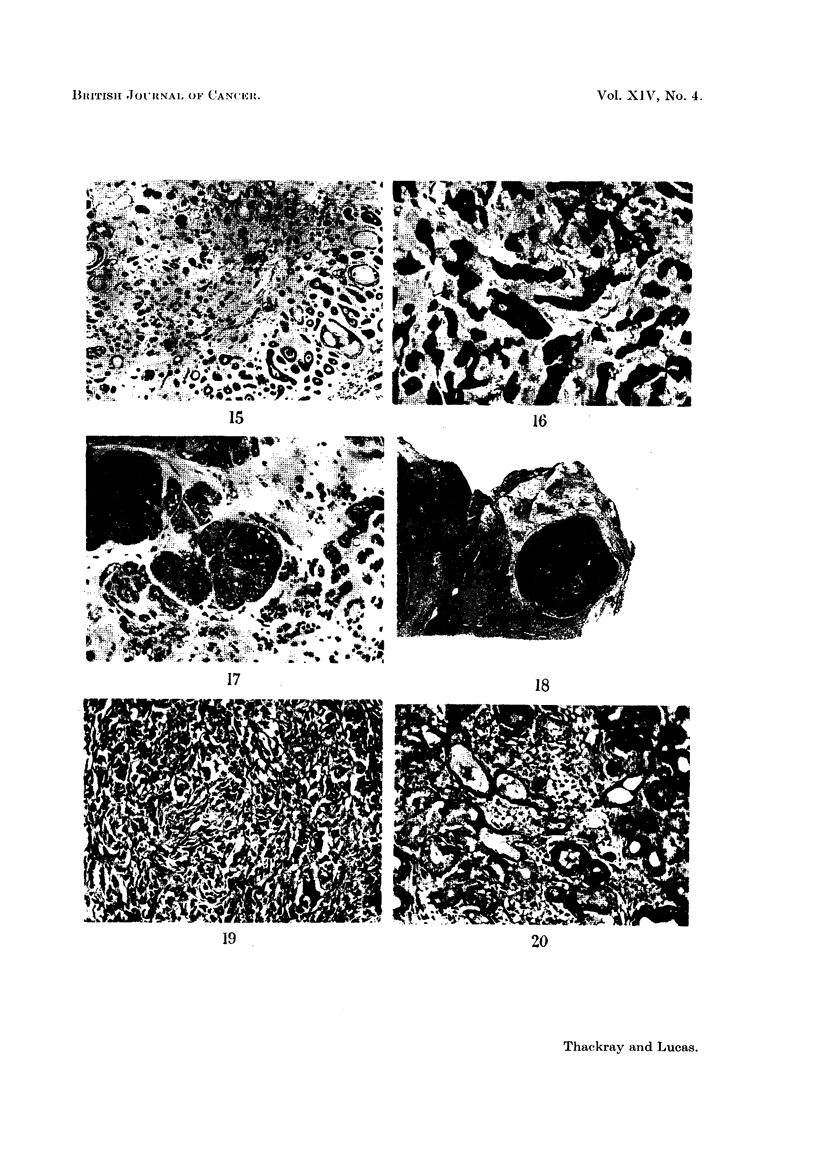# The Histology of Cylindroma of Mucous Gland Origin

**DOI:** 10.1038/bjc.1960.67

**Published:** 1960-12

**Authors:** A. C. Thackray, R. B. Lucas

## Abstract

**Images:**


### THE HISTOLOGY OF CYLINDROMA OF MUCOUS GLAND ORIGIN

A. C. THACKRAY AND R. B. LUCAS

*From the Bland-Sutton Institute of Pathology, Middlesex Hospital, London, W.1, and the Department of Pathology, Royal Dental Hospital of London School of Dental Surgery, London, W.C.2*

Received for publication October 27, 1960

It is now generally agreed that classification of mucous gland tumours on histological grounds is a useful procedure since the various types of tumour bear differing prognoses. Though there is still lack of unanimity with regard to details of nomenclature, most pathologists and clinicians would probably agree to the recognition of the following neoplasms as distinct entities.

1. *Mixed tumours.*—These are the neoplasms traditionally described as " mixed salivary tumours ". Typically, they consist of an epithelial component intimately associated with mucoid or myxochondroid intercellular material.
2. *Mucoepidermoid tumours.*—The neoplasms described by Stewart, Foote and Becker (1945), consisting of epidermoid and mucus-secreting elements.
3. *Cylindromas.*—Tumours of characteristic architecture, to be described in this paper.
4. *Glandular, squamous and other carcinomas.*—Frank carcinomas, mostly glandular, sometimes squamous and occasionally of other types, comparable in behaviour to carcinomas elsewhere.

These four groups of neoplasms constitute the great majority of all tumours of mucous glands and warrant separate recognition on grounds of structure and behaviour. In addition, there are the less common tumours such as those of the oxyphil, acinic cell and papillary cystadenoma type, and the mesenchymal tumours, which are not further considered here.

With regard to the behaviour of the commoner neoplasms, the mixed tumours are generally regarded as benign. However, they show a marked propensity for recurrence, due to factors such as the leaving behind of peripheral nodules of growth after enucleation, the implantation of neoplastic cells in the tumour bed and the presence of focal infiltrations (Patey and Thackray, 1958). Apart from recurrences due to such causes there is the question of whether mixed tumours can change in type to become carcinomatous. It is generally believed that such changes do occur in a certain proportion of cases (Foote and Frazell, 1953; Frazell, 1954; Patey and Thackray, 1958) but that on the whole they are infrequent.

The muco-epidermoid tumours, on the other hand, behave as malignant growths, both with regard to local invasion and metastatic deposits. The cylindromas show a similar natural history, for they too are locally invasive growths and are also capable of giving rise to metastases. Their rate of growth, however, is generally rather slow and they tend towards multiple recurrences before finally metastasizing. The frank carcinomas behave more aggressively than the tumours just mentioned, running a course more like carcinoma elsewhere.

It is evident, therefore, that accurate histological diagnosis is essential for the prognosis and treatment of mucous gland tumours. The purpose of this paper, based on the study of 42 tumours, is to deal in detail with the histopathology of one type of these neoplasms, the cylindroma, and to indicate the wide variety of appearances which may be encountered.

### *Nomenclature*

Billroth in 1859 gave the first account of a cylindroma. In his original case the neoplasm had invaded the orbit, probably from the mucous glands of the accessory nasal sinuses and he coined the name cylindroma because the epithelial elements of the growth were enclosed in well-defined " cylinders " of connective tissue. Malassez (1883) mentioned some earlier cases that appeared to fall into the same group. The term " basalioma " was first used by Krompecher (1908), who considered the tumour to be of analogous nature to the basal cell growths of the skin. Ahlbom (1935) and Ringertz (1938) also used the term basalioma. In the more recent American literature the tumour has generally been known as " adenoid cystic carcinoma ", a term apparently introduced by Ewing (Foote and Frazell, 1953), and series of tumours under this name have been reported by Spies (1930) and others. Spies emphasized that the tumour was a distinct entity and should not be confused with the adenoid cystic epithelioma of Brooks and Fordyce, and that it was different from adenocarcinoma. Adenocarcinoma of mixed tumour type has also been used as a designation (New and Childrey, 1931 ; Watson, 1935), and adenocarcinoma of cylindroma type (Dockerty and Mayo, 1942, 1943 ; Quattelbaum, Dockery and Mayo, 1946). The original term cylindroma has come back into favour recently, and Lennox (1960) comments " I can find no better name than ' cylindroma ' for the group, though few members contain any cylindrical structures ".

Tumours of cylindromatous type were at first regarded simply as variants of the " benign salivary gland tumour ", as it was often called, and were not considered to differ in any significant manner from the majority of such tumours. Nevertheless, even early reports had shown that these neoplasms were very apt to recur after local removal, and could even metastasize (Ribbert, 1907). On the other hand, some tumours which behaved in a malignant manner were thought of as malignant mixed tumours rather than the cylindromas they undoubtedly were, owing to inadequate appreciation of the structural distinctions between the two types of tumour. Later, however, the structural characteristics of the cylindroma came to be understood, and also a general appreciation of their clinical behaviour. Montanus (1938) and Mulligan (1943) review the earlier reports of metastasizing tumours of the salivary glands, many of which were undoubtedly cylindromas.

### *Distribution*

It has been recognized for many years that as well as occurring in the major salivary glands tumours of cylindromatous type may also be found in the minor salivary glands within the oral cavity and in mucous glands elsewhere. Malassez

(1883) was one of the earliest authors to describe a tumour of the palate as cylindroma-like. A large number of reports of palatal mucous gland tumours have since appeared though the neoplasms are very often reported as " mixed " or " benign salivary " tumours. The literature of these earlier accounts may be found in Ringertz (1938). More recent reports of intra-oral salivary gland tumours have given some prominence to the question of histological types and most authors have stressed the necessity for distinguishing between " mixed " tumours and the other varieties of mucous gland neoplasms. Recent series are those of Rawson, Howard, Royster and Horn (1950), Russell (1955), Ranger, Thackray and Lucas (1956) and Harrison (1956, 1957). The intra-oral glands are the most frequent site of occurrence of cylindroma for though salivary gland tumours in general occur less commonly in the minor salivary glands the proportion of cylindromas occurring in those glands is about 15 per cent of all tumours (Ranger, Thackray and Lucas, 1956), whereas in the major salivary glands it is in the region of 4 per cent (Foote and Frazell, 1953). In the case of the major glands the parotid and submaxillary are chiefly affected, cylindromas of the sublingual gland being very rare. Intra-orally, the glands of the palate and the floor of the mouth are the main sites, about 70 per cent of all intra-oral cylindromas occurring in these two areas. The remaining tumours occur in the tongue, the lip, or elsewhere in the buccal mucosa.

Cylindroma also occurs in the respiratory tract with some frequency. In fact, in a large series of tumours from all sites there are likely to be more neoplasms from the respiratory tract, including the nose, naso-pharynx and sinuses, than from the salivary glands. However, it is only comparatively recently that cylindroma of the respiratory glands has been recognized as a distinct entity. Kramer and Som (1939) were the first to report a tumour of this type occurring in the bronchus, and they give the early literature for the tumour in other parts of the respiratory tract. Subsequently there have been numerous reports of such lesions, most authors giving prominence to the question of differentiating the various types of neoplasm that can arise from the respiratory glands. It is now generally agreed that a clear distinction should be made between cylindroma, bronchial adenoma of the carcinoid type and frank bronchial carcinoma (McDonald and Havens, 1948 ; Belsey and Valentine, 1951 ; Reid, 1952), though occasionally tumours occur which appear to share characteristics both of the solid adenoma and the cylindroma (Englebreth-Holm, 1944). In Enterline and Schoenberg's (1954) series, bronchial cylindromas proved ultimately fatal seven times as frequently as " carcinoid " adenomas, and also recurred seven times as frequently.

Cylindroma also occurs in the lachrymal gland, where it constitutes some 50 per cent of all tumours, the other 50 per cent being of the mixed type (Godtfredsen, 1948). Occasional breast tumours show the cylindromatous pattern (Stewart, 1946), as do a number of skin tumours deriving from the sweat glands (Lever, 1948).

### *Histology*

*General histological pattern.*—The most commonly observed histological pattern in cylindroma is that shown in Fig. 1, the tumour consisting of irregularly shaped masses of cells in a rather scanty connective tissue stroma. Numerous cystic or alveolar spaces are present in the cell masses, giving rise to the cribriform effect which is a very characteristic feature of this neoplasm. However, not all tumours show the cribriform pattern since the cystic spaces are sometimes absent. In such cases, the growth is of a solid type. Variations of the cystic or the solid pattern account for much of the variation to be observed in the general histological picture in cylindroma, though changes in the stroma also contribute in some cases.

*The cystic pattern.*—The cystic spaces in a cylindroma are not all formed in the same manner. In Fig. 2, for example, the space represents a duct, its wall consisting of a double row of cells arranged in a similar manner to those of a normal salivary gland duct. The cells of the inner row, which will be referred to as lumenal cells, are small, with oxyphilic cytoplasm and a vesicular nucleus which contains a prominent nucleolus. These lumenal cells probably also have some secretory activity. The cells of the outer row are larger, though the nucleus is smaller than those of the inner row. The cytoplasm is markedly vacuolated and the nucleus stains darkly. Nucleoli are rarely seen. These cells are similar in appearance to the myoepithelial cells of the normal duct. The material within the lumen is granular and shows well-marked eosinophilia in sections stained by a routine haematoxylin and eosin method, and it also stains very strongly with the periodic acid-Schiff technique.

The occurrence of cystic structures with the two rows of cells regularly disposed around the lumen in the manner just described is the exception rather than the rule, for in the great majority of cases one or other cell type preponderates. Thus in Fig. 3 the cystic structure towards the lower part of the field is lined by cells of the myoepithelial type whilst most of the other cysts are lined only by cells of the lumenal type. A point of note is the difference between the contents in these two types of cyst. In those lined by myoepithelial cells the contents are not markedly eosinophilic and are only weakly P.A.S.-positive.

Apart from arrangements of the types just mentioned, where the cysts are bounded by one or other of the cell types alone and apart also from those instances in which there is a regular arrangement of the cells in two layers, as in Fig. 2, various other configurations may also be encountered. Thus in Fig. 4 the large duct-like structure in the centre of the field is lined by a single layer of lumenal cells, while the myoepithelial cells next to this layer are present in sheets in the lower part of the field. On the other hand, above the duct, these cells have undergone apparently degenerative changes and only cell remnants in a mucoid matrix are to be seen. In some cases the myoepithelial cells predominate, with the presence of a good deal of mucoid intercellular material (Fig. 5).

As a further possibility there may be dilatation of the duct-like spaces rather than proliferation of cells. When this proceeds to an extreme extent, the lace-like pattern shown in Fig. 6 results. In other cases the cystic dilatation leads to the formation of structures which are much more duct-like in appearance (Fig. 7). Another expression of duct formation is that illustrated in Fig. 8. Here the tumour consists almost entirely of compressed ductular structures lined by a double row of cells disposed in a very regular manner.

Cyst-like spaces may also result from the enclavement of areas of stroma, or of mucoid material which is produced by the tumour cells in proximity to the stroma. When such areas, because of the plane of section, appear to be enclosed by epithelium the resemblance to an epithelial cyst is close. However, serial sections show that such enclaved areas are due simply to the particular manner in which stroma and epithelium are juxtaposed. Thus in Fig. 10 the area at "A"

becomes " encircled " in further sections, by epithelium, assuming an appearance very similar to that seen at " B ". Furthermore, the contents of cystic spaces of this type are only very weakly P.A.S.-positive, in contradistinction to the spaces lined by lumenal cells (Fig. 9).

These variations in histological pattern, with the differing modes of cyst formation, account for the apparent discrepancies in the staining reactions of the mucoid material in the cystic spaces which have been noted by a number of workers (Lemaitre, 1938 ; Kramer and Som, 1939 ; MacDonald, Moersch and Tinney, 1945 ; Belsey and Valentine, 1951). Recently, however, Azzopardi and Smith (1959) have shown that there are, in fact, histochemical differences between the mucins in the different types of spaces. In the duct-like structures the mucin is strongly P.A.S.-positive and is not metachromatic, whilst in what they term the pseudo-acini (i.e., the spaces bordered by myoepithelial cells) the mucin is only weakly P.A.S.-positive but is strongly gamma-metachromatic, showing marked reduction of the metachromasia after incubation with hyaluronidase. The hyaline material which appears to be formed in proximity to the stroma is P.A.S.-positive to a moderate degree and is rather less gamma-metachromatic. Methylene blue extinction confirms these differences.

Comparable histochemical observations have been made in the case of mixed tumours. Some authors, for example Hemplemann and Womack (1942), have taken this to indicate that mixed tumours are of diploblastic origin, while others (Grishman, 1952) consider the connective tissue type of mucin to be produced by myoepithelial cells. The work of Erichsen (1955) and Cotchin (1958) on mammary neoplasms in the bitch provides additional evidence that mucin produced by myoepithelial cells is of what is generally considered to be connective tissue type.

*Solid patterns.*—Solid types of growth are much less common than the cribriform pattern and generally a part only of the growth is arranged in this manner, the remainder being of the more usual architecture. However, occasionally an almost completely solid type of growth is encountered. Such growths may be difficult to identify, though thorough examination will generally reveal small areas here and there exhibiting more characteristic traits. Compression of ductular structures, as in Fig. 8, may lead to a practically solid type of growth, but more often the appearances are due to the tumour cells forming continuous sheets. Areas of necrosis tend to occur in solid growths (Fig. 11). Mitotic figures do not occur with any frequency in either solid or cystic tumours.

Two further uncommon variants may be mentioned. In one the cells are predominantly fusiform or spindle-shaped, the appearance in some fields being reminiscent of a neurinoma, with what appears to be palisading of the nuclei (Fig. 12 and 13). In the other the bulk of the tumour is composed of lumenal cells in an acinar arrangement. Only in a few areas at the edge of the tumour are myoepithelial cells to be seen grouped around the tubules and giving a clue to the cylindromatous nature of the growth (Fig. 14).

*Stromal changes.*—Stromal changes are sometimes prominent in cylindroma. A rather scanty fibrous stroma is the commonest finding (Fig. 1) but often the stroma is more abundant and frequently hyaline is present. The hyaline is either changed stromal connective tissue or it represents a product of the tumour cells, being laid down in proximity to the stroma. Hyalinization is often associated with a breaking-up of the cribriform pattern to form smaller cell groups

(Fig. 15). The deposition of mucinous material in proximity to the stroma, or the replacement of the stroma itself by mucoid, is also not uncommon. However, the tumour cell masses generally remain sharply defined and do not merge with the mucoid material, as occurs so frequently, and typically in mixed tumours. Thus in Fig. 16 the cell groups have quite distinct outlines, and are clearly demarcated from the stroma, which is almost entirely mucoid. Occasionally, however, there are encountered tumours in which the epithelial cells appear to " melt " into the mucoid in much the same way as occurs in mixed tumours. This is shown in Fig. 17, and much of the tumour from which this section was prepared showed similar changes.

Cyst formation in the stroma has already been discussed.

*Differentiation between cylindroma and mixed tumour.*—In the great majority of cases there is no difficulty in differentiating between typical examples of cylindroma and typical mixed tumours. Occasionally, however, instances occur where the distinction is not so readily made. One source of possible confusion is the mucoid change which is a typical feature of the mixed tumour and sometimes also occurs in the cylindroma. However, the distinguishing feature, as already pointed out, is the clear demarcation of epithelial cells from mucoid intercellular material in cylindroma, whereas in mixed tumour the epithelial cells appear to blend with the mucin. The occasional cylindroma in which mucoid intercellular change appears also to involve the epithelial cells, as shown in Fig. 17, may be confused with mixed tumour. A very unusual example of this type of mucoid change in a cylindroma is shown in Fig. 18. The tumour, from the parotid, showed on macroscopic examination a small nodule of different appearance to the rest of the growth, measuring just under 1 cm. in diameter and situated at one pole. On section the greater part of the tumour showed the typical cribriform pattern of cylindroma but the structure of the nodule, on preliminary examination, appeared very similar to that of a mixed tumour, consisting of scattered groups of epithelial cells and ductular structures in a completely mucoid matrix. Serial sections of the entire area showed the nodule to be clearly demarcated by a distinct fibrous capsule from the rest of the tumour, with which no connection could be demonstrated. However, more detailed examination showed the cells to be of typical cylindromatous type and clearly demarcated from the surrounding mucoid material.

Another type of configuration in cylindroma that shows some similarity to that seen in mixed tumour is the pattern illustrated in Fig. 5. This is comparable to those mixed tumours in which the cells tend to be of stellate shape. Foote and Frazell (1953) refer to such areas in mixed tumours as " pseudo-adenoid cystic ", and found them in about 10 per cent of their cases, usually in limited amount. Duct formation in cylindroma (Fig. 7) may also produce appearances very similar to that seen in some mixed tumours.

In a small number of cases it may be impossible to decide whether a given tumour should be classified as mixed or as cylindroma.

*Relationship to spheroidal cell carcinoma.*—Ringertz (1938) and some other writers thought it possible to distinguish between benign and malignant cylindromas, those growths in which cyst formation was evident tending to belong to the former class and the solid tumours to the latter. Subsequent studies, however, have not shown any correlation between histological pattern and behaviour. The majority of tumours, whatever the histological picture, follow the same type of course. This tends to be rather prolonged and is characterized by slow but persistent infiltration, growth along nerves often being a prominent feature. Metastases occur relatively late in the course of the disease and the secondary deposits generally show the same histological appearance as the primary growth. The same applies to local recurrences, even after a number of years. In some cases, however, local recurrences or metastases may show quite a different picture, presenting as spheroidal cell carcinoma from the histological point of view, and clinically assuming a more frankly carcinomatous course. Fig. 19 is an example of this type of growth. The primary growth, in the trachea, showed the typical cribriform pattern. The section illustration was from a local recurrence.

### *Histogenesis*

Theories of origin of mucous gland tumours in general have been dealt with extensively in the literature, but there is very little with regard to cylindroma in particular.

An early view was that of Krompecher (1908), who considered the tumour to be akin to the basal cell carcinoma of the skin, both clinically and pathologically, and for this reason he used a corresponding nomenclature. Other authors have also referred to cylindroma as basal cell carcinoma or basalioma, either because such terms have appeared descriptively appropriate or because they have attributed a mucosal basal cell origin to the growth (Beck and Guttman, 1936). However, most authors who deal with the question of origin are agreed that the cylindroma arises directly from mucous, salivary or other glands, though there is some doubt as to which elements of the glands are responsible. This view is supported by the finding of tumour originating, or apparently originating, from gland elements, though reports of this are remarkably scanty, considering the number of tumours recorded in the literature. However, McDonald, Moersch and Tinney (1945) state that in one of their six cases the tumour appeared to be arising from mucous glands in the bronchial wall and Reid (1952) has also seen tumour originating in the duct of a bronchial gland. Russell (1955) suggests that the tumour arises or appears to arise from dilated mucous gland ducts.

In the neoplasms reported in this study, the close proximity of tumour to relatively normal mucous glands was noted in a number of cases. Mostly there was a clear demarcation between tumour and normal tissue, but sometimes growth and gland were intermingled. In such cases it might well have been the case that normal glandular tissue was being invaded by tumour, rather than giving rise to it, though in one lesion there appeared to be an actual transition between tumour tissue and normal gland.

The so-called " transitional lobules " have been thought to constitute transitional stages between normal glandular tissue and tumour. These lobules are aggregations of glandular tissue showing some dilatation of the ducts and degenerative changes in their lining cells and in the cells of adjacent alveoli. Chronic inflammatory infiltration is present in the stroma. These changes have been described in connection with mixed tumours though not so far as can be ascertained in cylindroma. Fig. 20 shows an example of this type of change in one of our cases, but we agree with Ringertz (1938) that the changes are degenerative and not neoplastic.

The expenses of this investigation have been defrayed by the British Empire Cancer Campaign.

### REFERENCES

AHLBOM, H. E.—(1935) *Acta Radiol.*, Suppl. 23.
AZZOPARDI, J. G. AND SMITH, O. D.—(1959) *J. Path. Bact.*, **77**, 131.
BECK, J. C. AND GUTTMAN, M. R.—(1936) *Ann. Otol., etc., St. Louis*, **45**, 618.
BELSEY, R. H. R. AND VALENTINE, J. C.—(1951) *J. Path. Bact.*, **63**, 377.
BILLROTH, T.—(1859) *Virchows Arch.*, **17**, 357.
COTCHIN, E.—(1958) *J. comp. Path.*, **68**, 1.
DOCKERTY, M. B. AND MAYO, C. W.—(1942) *Surg. Gynec. Obstet.*, **74**, 1033.—(1943) *Surgery*, **13**, 416.
ENGELBRETH-HOLM, J.—(1944) *Acta chir. scand.*, **90**, 383.
ENTERLINE, H. T. AND SCHOENBERG, H. W.—(1954) *Cancer*, **7**, 663.
ERICHSEN, S.—(1955) *Acta. path. microbiol. scand.*, **36**, 490.
FOOTE, F. W., JR. AND FRAZELL, E. L.—(1953) *Cancer*, **6**, 1065.
FRAZELL, E. L.—(1954) *Ibid.*, **7**, 637.
GODTFREDSEN, E.—(1948) *Brit. J. Ophthal.*, **32**, 171.
GRISHMAN, E.—(1952) *Cancer*, **5**, 700.
HARRISON, K.—(1956) *Ann. R. Coll. Surg. Engl.*, **18**, 99.—(1957) *Ann. Otol., etc., St. Louis*, **66**, 459.
HEMPLEMANN, L. H., JR. AND WOMACK, N. A.—(1942) *Ann. Surg.*, **116**, 34.
KRAMER, R. AND SOM, M. L.—(1939) *Arch. Otolaryng., Chicago*, **29**, 356.
KROMPECHER, E.—(1908) *Beitr. path. Anat.*, **44**, 88.
LEMAITRE, Y.—(1938) *Ann. Oto-laryng.*, **57**, 185.
LENNOX, B.—(1960) in Recent Advances in Pathology ', 7th Ed., edited by C. V. Harrison. London (J. & A. Churchill), p. 9.
LEVER, W. F.—(1948) *Arch. Derm. Syph., N.Y.*, **57**, 332.
MCDONALD, J. R. AND HAVENS, F. Z.—(1948) *Surg. Clin. N. Amer.*, **28**, 1087.
*Idem*, MOERSCH, H. J. AND TINNEY, W. S.—(1945) *J. thorac. Surg.*, **14**, 445.
MALASSEZ, M. L.—(1883) *Arch. Phys. norm. path.*, 3me série, **1**, 186.
MONTANUS, W. P.—(1938) *Surgery*, **4**, 423.
MULLIGAN, R. M.—(1943) *Arch. Path. (Lab. Med.)*, **35**, 357.
NEW, G. B. AND CHILDREY, J. H.—(1931) *Arch. Otolaryng., Chicago*, **14**, 669.
PATEY, D. H. AND THACKRAY, A. C.—(1958) *Brit. J. Surg.*, **45**, 477.
QUATTLEBAUM, F. W., DOCKERTY, M. B. AND MAYO, C. W.—(1946) *Surg. Gynec. Obstet,.* **82**, 342.
RANGER, D., THACKRAY, A. C. AND LUCAS, R. B.—(1956) *Brit. J. Cancer*, **10**, 1.
RAWSON, A. J., HOWARD, J. M., ROYSTER, H. P. AND HORN, R. C., JR.—(1950) *Cancer*, **3**, 445.
REID, J. D.—(1952) *Ibid.*, **5**, 685.
RIBBERT, M. W. H.—(1907) *Dtsch. med. Wschr.*, **33**, 126.
RINGERTZ, N.—(1938) *Acta Otolaryng., Stockh.*, Suppl. 27.
RUSSELL, H.—(1955) *Brit. J. Surg.*, **43**, 248.
SPIES, J. W.—(1930) *Arch. Surg., Chicago*, **21**, 365.
STEWART, F. W.—(1946) *Surgery*, **19**, 74.
*Idem*, FOOTE, F. W. AND BECKER, W. F.—(1945) *Ann. Surg.*, **122**, 820.
WATSON, W. L.—(1935) *Amer. J. Roentgenol.*, **34**, 53.

### EXPLANATION OF PLATES

FIG. 1.—A commonly occurring pattern in cylindroma. The tumour consists of cell masses containing cystic spaces, giving rise to a cribriform appearance. ×60.

FIG. 2.—High power view of a cylindroma, showing the two cell types arranged as in a normal mucous gland duct. ×330.

FIG. 3.—To show the two types of cellular arrangement around cystic spaces. ×250.

FIG. 4.—A duct lined by lumenal cells at the centre of the field, with myoepithelial cells above and below. ×330.

FIG. 5.—The cells in this field are nearly all of myoepithelial type, with a considerable amount of intercellular mucoid material. ×95.

FIG. 6.—A typical appearance in cylindroma ; well-marked cribriform pattern. ×45.

FIG. 7.—To show the formation of rather irregular duct-like spaces. ×95.

FIG. 8.—To show compressed duct-like structures. ×95.

FIG. 9.—Darkly stained contents of lumenal cysts contrasted with weakly P.A.S.—positive stromal cysts. ×185.

FIG. 10.—Mode of formation of stromal cysts. " A " indicates an area of stroma about to become enclaved by epithelium ; " B " indicates a similar area, completely encircled. ×185.

FIG. 11.—Area of necrosis in a predominantly solid growth. ×60.

FIG. 12.—Bundles of elongated cells reminiscent of a neurinoma in the upper part of the field, with more typical cylindromatous pattern below. ×100.

FIG. 13.—High power view of the spindle cell area in Fig. 12. ×190.

FIG. 14.—Field at the edge of a tumour which for the most part was composed of cells of lumenal type. ×190.

FIG. 15.—Breaking-up of the cribriform pattern associated with hyalinisation. ×45.

FIG. 16.—Sharply defined cell groups with intervening mucoid stroma. ×95.

FIG. 17.—Epithelial cells in a cylindroma appearing to melt into the mucoid. ×95.

FIG. 18.—Distinct nodule of growth in a parotid cylindroma. ×3.

FIG. 19.—Anaplasia in the local recurrence of a formerly well differentiated cylindroma. ×95.

FIG. 20.—A " transitional " lobule of mucous glands, adjacent to a cylindroma. ×95.